# A Retrospective Study after 10 Years (2010–2019) of Meat Inspection Activity in a Domestic Swine Abattoir in Tuscany: The Slaughterhouse as an Epidemiological Observatory

**DOI:** 10.3390/ani10101907

**Published:** 2020-10-18

**Authors:** Lisa Guardone, Alessio Vitali, Filippo Fratini, Stefano Pardini, Beniamino Terzo Cenci Goga, Daniele Nucera, Andrea Armani

**Affiliations:** 1 Department of Veterinary Sciences, University of Pisa, Viale delle Piagge 2, 56124 Pisa, Italy; lisa.guardone@for.unipi.it (L.G.); alessiovitali21@gmail.com (A.V.); filippo.fratini@unipi.it (F.F.); 2 Centro Interdipartimentale di Ricerca Nutraceutica e Alimentazione per la Salute, Nutrafood, Università di Pisa, 56124 Pisa, Italy; 3 Azienda USL Toscana Centro, zona Val di Nievole, via 1 Maggio Massa e Cozzile, 51010 Pistoia, Italy; stefano.pardini@uslcentro.toscana.it; 4 Department of Veterinary Medicine, University of Perugia, Via San Costanzo 4, 06126 Perugia, Italy; beniamino.cencigoga@unipg.it; 5 Department of Agriculture, Forest and Food Science, University of Turin, Largo Braccini 2, 10095 Torino, Italy; daniele.nucera@gmail.com

**Keywords:** slaughter, pathology, erysipelas, carcass condemnation, welfare

## Abstract

**Simple Summary:**

Veterinarians belonging to the Health Authorities are responsible for the safety of food of animal origin. The control activities performed at the slaughterhouse comprise inspections before and after slaughtering to verify the health and welfare of the animals and the wholesomeness of the animal products. In this study, data deriving from ten years (2010–2019) of meat inspection activity in a pig slaughterhouse in Tuscany (Italy) were analyzed to investigate the frequencies of death during transport of whole carcass and partial condemnations and their causes. In total 1,246,309 pigs from 8 different regions of Central and Northern Italy were slaughtered. The mortality rate was found to be lower than the threshold internationally recommended to ensure animal welfare and in line with the mortality values reported at the European level. Overalls 372 carcasses were condemned mainly due to erysipelas, generalized jaundice, lipomatous pseudohypertrophy, generalized abscesses, acute or generalized enteritis, and peritonitis. As regards partial condemnations, liver, lungs, and kidney were the most frequently condemned organs. The results of this study describe a non-worrying situation as regards to the investigated aspects and confirm the slaughterhouse as a privileged observation point for monitoring the trend of the main diseases over time, the results of the efforts for their control, and also the compliance with animal welfare standards.

**Abstract:**

The activities performed by the Official Veterinarian at the slaughterhouse represent a useful source of data for the control of issues affecting human and animal health and welfare. This study analyzed the data deriving from ten years (2010–2019) of meat inspection in a pig slaughterhouse in Tuscany (Italy) to investigate the transport mortality rate and the frequencies and main causes of whole carcass and partial condemnations. In total, 1,246,309 pigs were slaughtered from 8 different regions of Central and Northern Italy. Overall, 1153 pigs died during transport (mortality rate 0.09%). Whole carcass condemnation affected 372 carcasses (0.03%), mainly due to erysipelas, generalized jaundice, lipomatous pseudohypertrophy, generalized abscesses, acute or generalized enteritis, and peritonitis. As regards partial condemnations, the liver was the most frequently condemned (~30% of the pigs), followed by lungs (17.3%), heart (6.9%), and kidney (0.9%). The main causes were “milk spot liver” and perihepatitis for the liver; pneumonia and pleurisy for the lungs; pericarditis and polyserositis for the heart; and polycystic kidney and nephritis for kidneys. The results of this study describe a non-worrying situation as regards the investigated aspects and confirm the slaughterhouse as a valid epidemiological observatory for monitoring the trend of the main diseases over time, the results of the efforts for their control, and the compliance with animal welfare standards.

## 1. Introduction

World meat consumption is constantly increasing, reaching an average of about 43 kg per person/yearly, almost double than in the 1960s [1,2]. Pork and poultry meat are the most consumed worldwide, even though strong differences exist among different geographical areas, also due to livestock sector development, cultures, and climatic conditions [2]. In Europe, where the average meat consumption per person per year is 69.8 kg, it mainly consists of pork (34.6 kg) and poultry (23.1 kg), followed by beef (10.7 kg) and sheep (1.4 kg) meat [2]. Similarly, also in Italy, pork meat is one of the most appreciated meat categories, accounting for almost half (36.8 kg) of the total per person per year meat consumption (75 kg). In fact, pork meat is eaten both as fresh and processed, being one of the most important products deriving from the Italian livestock sector [3,4]. Remarkably, almost all of the over 11 million pigs slaughtered every year from 2010 to 2020 in Italy belong to the “heavy” category (about 160–170 kg live body weight) [3], mainly bred to produce Protected Designation of Origin (PDO) hams or other processed products [5].

In the European Union (EU), food safety is based on a synergy between competent authorities (CA) and food business operators (FBOs) [6,7]. While all FBOs have to ensure compliance with EU agri-food chain requirements in their daily activities, the CA carry out official controls to verify that FBOs act in accordance with EU legislation [8]. As regards the meat chain, meat inspection (MI) aims to protect consumers from exposure to meat-borne hazards and to ensure the safety and quality of meat products consumed domestically or exported [9]. Accordingly, in EU member states, slaughtering occurs in establishments approved by the CA, and MI has the primary aim of preventing and detecting public health hazards, such as foodborne pathogens or chemical contaminants. In such a system, animals (or part of them) that are identified as not fit for human consumption are removed (i.e., their carcasses and/or offal) from the food chain [10]. This approach has allowed over the years to keep the previously most common zoonoses under control, including tuberculosis, cysticercosis, trichinellosis, and others [11,12]. In the last decades, the aim of MI has expanded, pursuing a one health approach. Thus, additional public health issues, as well as animal health and welfare, meat quality, control of slaughter by-products, and protection of the environment, have been taken into consideration [10,11,12,13,14,15]. MI is performed by the official veterinary system, currently in agreement with the Regulation (EU) 2017/625 of the European Parliament and of the Council [7] and the Commission Implementing Regulation (EU) 2019/627 [16]. In detail, the tasks of MI consist of checking food chain information (FCI), ante-mortem inspection of live animals, and post-mortem inspection of carcasses and offal involving visual inspection, palpation, and incision of particular organs and lymph nodes.

In recent years, the MI approach has been questioned, considering that the procedures designed to detect previously common zoonotic diseases have minimal connection to current public health risks and because of possible carcass contamination [9]. In the three-year period, 2011–2013, the European Food Safety Authority (EFSA) produced six scientific opinions on public hazards linked to MI, covering the following animal species: domestic swine, poultry, bovine animals, domestic sheep and goats, farmed game, and domestic solipeds [10]. Specifically regarding domestic swine, EFSA concluded that palpation or incisions should be omitted in pigs subjected to routine slaughtering, because the risk of microbial cross contamination is higher than the risk associated with potentially reduced detection of conditions targeted by those techniques. Therefore, in 2014, the EU became the first supranational government in the world to request a visual-only inspection for all swine herds slaughtered in member states that met certain epidemiologic and animal rearing conditions [9,17,18]. However, official veterinarians shall proceed with additional *post-mortem* inspection procedures using incision and palpation of the carcasses and offal in cases of suspected risks (Art. 1 paragraph 2 of [18]). This approach is currently extended also to the other species (Art. 24 of [16]).

MI output, as a valuable approach for collecting epidemiological data on many diseases and animal welfare issues and for understanding the causes of the losses at the slaughterhouse has long been acknowledged [19,20,21]. This was recently remarked by various authors [12,22,23,24,25,26,27,28] as well as by the World Organisation for Animal Health (OIE) [29]. However, the study of Stärk et al. [12] highlighted a substantial lack of suitable and accessible published data arising from MI activities across Europe. According to Ghidini et al. and Harley et al. [24,30], consistent data on *post-mortem* lesions are lacking, in particular for pigs.

Therefore, also considering that the use of the slaughterhouse as an epidemiological observatory for an integrated approach to the food chain has been recently advocated [15,31,32], the aim of the present study was to analyze the data deriving from ten years of MI activity in a pig slaughterhouse in Tuscany (Italy), which receives animals from different regions of Central and Northern Italy, to investigate the transport mortality rate and the frequencies and main causes of whole carcass and partial carcass condemnations. Finally, the need for a harmonized system of data collection and sharing was briefly addressed.

## 2. Materials and Methods

### 2.1. Data Collection

A retrospective observational study was conducted in a small–medium-sized pig abattoir slaughtering more than 12,000 animals per year (~2400 animals per week) located in northern Tuscany (Central Italy). The slaughterhouse receives mainly heavy pigs (mostly hybrids of Landrace, Large White, and Duroc of about 160–170 kg live body weight), from several regions of the North and Centre of Italy. The study is based on the data collected during MI activities and recorded in the database of the local health authority (Azienda USL Toscana Centro) by the official veterinarians in the last 10 years (2010–2019). In the analyzed period, the legislative basis defining the official control activities in general and at the slaughterhouse were Regulation EC 882/2004 and Regulation EC 854/2004, respectively. Data were saved in Excel. The following information was present in the databases: lot number and number of animals per lot; code, name, and province of the farm of origin; date of *ante-mortem* inspection; date of slaughtering and of *post-mortem* inspection; number and causes of total (whole carcass) or partial (part of carcass/one or more organs) condemnations; and material condemned (for partial condemnations).

### 2.2. Data Analysis

Data were analyzed using Excel functions for calculating (i) transport mortality rate (number of dead pigs/pigs transported); (ii) number, absolute and relative percentage, and causes of total condemnations (expressed per year as well as overall statistics); (iii) number, absolute and relative percentage, and causes of partial condemnations (expressed per year as well as overall statistics), as well as the part of the carcass or organs condemned. In addition, the following information was also collected: number of animals slaughtered (per year or as a total count); and number of regions and provinces of origin and number of animals slaughtered in each of them.

### 2.3. Statistical Analysis

The chi-square test (significance for *p* < 0.05) was used to evaluate differences between the years, seasons, months, and geographical origin (region and province of the conferring farm) in terms of deaths during transport and whole carcass condemnations and, due to the larger amount of data, only between the years for partial condemnations. As regards the seasonal trend, this was calculated both among the four astronomical seasons (starting dates: spring, 20 March; summer, 20 June; autumn, 22 September; winter, 21 December) and also according to Nannoni et al. [27] (summer: from April to September; winter: from October to March). The analyses were conducted on proportions to correctly compare samples of varying sizes. For each proportion, the exact confidence interval was calculated according to the normal approximation method (Wald) or, in the case of small sample size (*n*), with the Wilson method (https://epitools.ausvet.com.au/ciproportion), as recommended by Brown et al. [33]. The Spearman correlation coefficient (rho) and the Pearson correlation coefficient (*r*) were used to assess the correlation between the number of deaths per year and the number of total and partial condemnations, as well as among the most condemned organs.

## 3. Results

### 3.1. Total Number of Pigs and Geographical Origin

**Figure 1:**
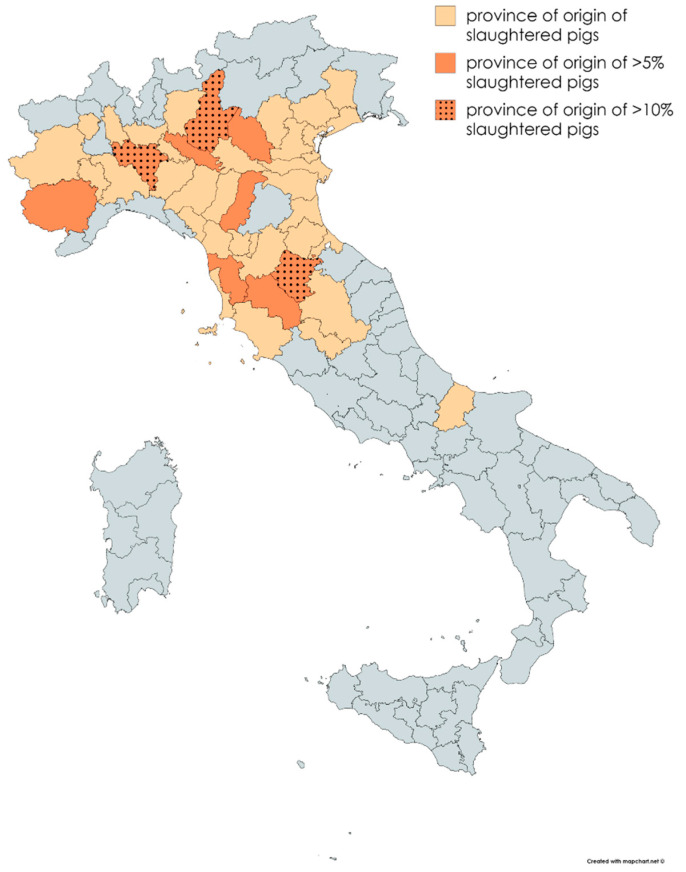
Provinces of origin of the pigs slaughtered in the examined period.

The plant slaughtered 124,631 heavy pigs on average per year (SD 8985.5). Details on the pigs conferred to the abattoir and slaughtered per year are reported in Table 1. The animals came from eight different regions of Central and Northern Italy, mainly Lombardy (*n* = 505,344, 40.51%), Tuscany (*n* = 365,972, 29.34%), and Emilia Romagna (*n* = 169,344, 13.57%), and to a lesser extent Veneto (*n* = 98,520, 7.90%), Piedmont (*n* = 92,856, 7.44%), Umbria (*n* = 13,609, 1.09%), Molise (*n* = 402, 0.03%), and Friuli Venezia Giulia (*n* = 262, 0.02%). More precisely, pigs came from 41 provinces of those regions (Figure 1) and three provinces in particular (Arezzo, Brescia and Pavia) contributed to approximately 38% of the total.

### 3.2. Deaths during Transport and Whole Carcass Condemnations

#### 3.2.1. Deaths during Transport

In the ten years analyzed, 1,153 animals (0.09% of the total number of the conferred animals, ranging from 0.02 to 0.26%) died during transport (Table 1). Statistically significant differences were observed for some years; in particular, the mortality rate observed in the years 2010, 2011, 2014, and 2015 appeared significantly higher than in the years 2012–2013 and 2016–2019. Detailed information on the transport date and on the geographical origin for pigs that died during transport were available only for the last 4 years (2016–2019); therefore, the reasons behind the observed significant differences could not be further investigated, and the seasonal and monthly trend could be analyzed only for this period. In the last 4 years, 236 pigs died during transport, contributing to a mortality rate of 0.05% (0.03–0.06%). Most of the deaths during transport (35.17%) were observed in summer, while only 16.53% of them occurred in winter (*p* = 0.0004). Spring and autumn showed comparable values (24.58% and 23.73%, respectively). Additionally, analyzing the data per year, summer was always the season with the highest number of deaths, although a statistically significant difference in comparison with the winter season was observed only for two years (2016–2017). No statistically significant difference was observed when applying the two seasons proposed by Nannoni et al. [27]. Overall, the months with a significantly higher number of deaths were August, September, and December. The highest mortality rate was observed for Piedmont and Lombardy regions (0.071% and 0.051%, respectively), while the province with the highest mortality rate was Florence (0.10%).

#### 3.2.2. Whole Carcass Condemnations

Overall, 372 carcasses (0.03% of the total number of slaughtered animals) were condemned. No correlation was observed between the deaths during transport and number of whole carcass condemnations per year. No statistically significant differences in relation to the absolute percentages of whole carcass condemnations in the various years were also observed. As regards the seasonal trend, a higher relative percentage of whole carcass condemnations was observed in winter and spring compared to autumn and summer (*p* = 0.02). The highest relative mortality rate was observed in May, but the lowest rates were observed in June and November; however, such differences were not statistically supported, considering the not significant difference between summer and fall. A significantly higher rate of whole carcass condemnation was observed for Emilia Romagna region and for Vicenza and Pistoia provinces.

The main causes of whole carcass condemnations, accounting for over 60% of them, were erysipelas (37.36%) and generalized jaundice (26.07%). Other causes accounting for more than 5% each were lipomatous pseudohypertrophy (9.95%), abscesses (7.53%), and enteritis (5.38%). The remaining causes are reported in detail in Table 2. A statistically significant higher frequency was observed for erysipelas in the years 2016, 2017, and 2019 and in 2018 for generalized jaundice.

### 3.3. Partial Condemnations

The total number of organs condemned was 641,818. It should be specified that this figure does not correspond precisely to a number of pigs, as different organs condemned from the same subject were recorded separately. When partial condemnations did not indicate a single organ but were reported as pluck, thoracic viscera, or abdominal viscera, the term pluck was attributed to heart, lungs, and liver; thoracic viscera to heart and lungs; and abdominal viscera to liver, spleen, and the remaining abdominal viscera. The organs most frequently condemned were the liver (30.89% of the slaughtered pigs), followed by lungs (17.26%), heart (6.94%), and kidney (0.95%) (Table 3). No correlations were observed among the frequency of condemnation of the above-mentioned organs.

The main causes of condemnation were so-called “milk spot liver” (or white spot liver) (91.7% of the condemned livers) and perihepatitis (6.92%) for the liver; pneumonia (including bronchopneumonia and pleuropneumonia) (69.2% of the condemned lungs) and pleurisy (24.56%) for lungs; pericarditis (66.63% of the condemned hearts) and polyserositis (20.88%) for the heart; and polycystic kidney disease (64.92% of the condemned kidneys) and nephritis (33.09%) for kidney. Other organs condemned, to a much lesser extent, were heads (0.002%, *n* = 23) and hams (0.003%, *n* = 33) due to the presence of abscesses.

## 4. Discussion

### 4.1. Total Number of Slaughtered Pigs and Geographical Origin

According to the National Statistical Institute, in Italy between 2010 and 2019, 12,153,392 pigs were slaughtered yearly, on average [3], in 707 slaughterhouses [34]. The Italian pig production is mainly concentrated in the northern part of the country. In fact, in 2019, 9,403,054 pigs were slaughtered in the North, representing 82.48% of the total Italian production, while 762,834 and 1,234,702 pigs were slaughtered in the Center and in the South, respectively, accounting for 6.69% and 10.83% of the national overall, respectively. In 2019, 125,845 pigs were slaughtered in the examined slaughterhouse, corresponding to around 17% of the pigs slaughtered in the Center. Heavy pigs represented the most slaughtered category throughout the national territory, although the slaughtering of “light” pigs is to some extent more widespread in the South than in the North and Centre [35]. While Southern Italy is characterized by many small-scale farms and many low productivity slaughterhouses, in the North of Italy, large-scale indoor intensive farms and high production slaughterhouses are present [24].

The geographical origin (see Section 3.1) of the heavy pigs slaughtered in the abattoir analyzed in this study could be explained considered that the establishment also includes a cutting plant where Protected Designation of Origin (PDO) products are produced. Among the most renowned Italian PDO products deriving from pork meat are the “Prosciutto di Parma”, “Prosciutto Toscano”, and “Prosciutto di San Daniele” [36]. Their product specifications [37,38,39] establish the geographical area of origin of the pigs, indicating an area comprising the whole north and a large part of the center of Italy, including Emilia-Romagna, Lombardy, Marche, Umbria, Tuscany, and Lazio for all three of them, and also Piedmont, Veneto, Abruzzo, and Molise for Prosciutto di Parma and Prosciutto di San Daniele (for the latter, pigs from Friuli Venezia Giulia are also included). In fact, all the slaughtered pigs came from regions included in the aforementioned list, with the only exception being Lazio, from which no animals were conferred.

### 4.2. Deaths during Transport

The European legislation on the protection of animals during transport and related operations aims at guaranteeing animal welfare and places as a general condition that “*No person shall transport animals or cause animals to be transported in a way likely to cause injury or undue suffering to them*”. In addition, it establishes that a series of conditions, including the suitability of the animals for transport and the specific training of operators [40], must be met. Transport mortality also represents an economical issue for producers due to the loss of revenues. In fact, according to the current European legislation, meat should be declared unfit for human consumption if it comes from animals that died before being slaughtered (Art. 45 [16]). Furthermore, injuries, fractures, and bruises, which can occur during transport and in loading and unloading operations, can compromise the meat quality, in particular of valuable cuts such as ham, loin, and shoulder [41].

The transport of live pigs remains a critical point in the European pig meat chain [42,43]. The same issue has been reported in third countries [41,44]. In fact, although transport represents a short phase compared to the total duration of the production cycle, it involves a wide variety of highly stressful elements [43,45]. Loading and unloading are particularly critical moments [41]; mixing up different groups of animals can also cause severe stress and induce aggressive behavior [46,47]. In addition, the loading density and time, the truck structure, the environmental temperatures, the lack of a fasting period before transport, and the duration of transport may influence the welfare [27,42].

Thus, the transport mortality rate represents a simple, objective, and useful indicator of animal welfare during transport [48]. According to internationally recognized animal welfare experts, mortality should remain below the 0.1% threshold [49]. The rate found in this study (0.09%), where all the animals were conferred to the slaughterhouse in 5–6 h (author’s note), is below this threshold and in line with the mortality values of 0.03–0.5% reported at the European level for transport within 8 h [42,43] and with the average mortality value (0.11%) found in the study of Averós et al. [42] that analyzed 739 trips transporting pigs to 37 slaughterhouses in five member countries (Spain, Portugal, France, Italy, and Germany) between June 2003 and May 2004. However, it should be specified that these last data refer to the period prior to the entry into force of the Council Regulation (EC) No 1/2005, on the protection of animals during transport [40]. A slightly lower value is reported by a more recent Portuguese study of Garcia-Diez et al. (0.09% [30]), which observes a reduction in transport mortality associated with the compulsory certification of vehicles and drivers according to Council Regulation (EC) No 1/2005 [40]. Lower values (~0.05%) are reported in a Spanish and an Italian study [27,50], while no transport mortality was observed by Maisano et al. [51] in Italy.

As regards the seasonal mortality, the higher rate observed in summer (astronomically defined) and in August, September, and December seems to confirm the sensitivity of pigs to thermal stress [27,30,48,52,53]. In fact, although the actual temperatures of the investigated years were not collected, the average temperature in August, September, and December in the North and Centre of Italy are outside the thermal neutral range for pigs (15–25 °C) [30].

Finally, it should be noted that all the above-mentioned problems can be observed to be not only associated with long distances, but also during short transports, especially in the case of high temperatures and genetic predisposition [41,54], in particular for heavy pigs [48], such as those analyzed in this study. In fact, the province with the highest mortality rate was Florence, one of the closest to the province hosting the investigated slaughterhouse. The reason for this is difficult to speculate, as deaths were distributed in different months and, although all the animals came from the same farm, this conferred over 80% of the pigs from the province.

### 4.3. Whole Carcass Condemnations

The overall percentage of condemned carcasses out of the total slaughtered pigs (0.03%), which was constant over the years, is significantly lower compared to partial condemnations, as already reported in a previous study of Ceccarelli et al. [22]. Higher percentage values (0.17% and 0.12%) are reported in two Italian studies [22,51], which collected data in a slaughterhouse in Central and Northern Italy, respectively. However, it should be specified that the rate reported by Maisano et al. [51] considers not only the carcasses condemned at the post-mortem visit, but also the animals killed during transport and emergency slaughter. Therefore, it is more correct to compare this value with the percentage (0.12%) obtained adding deaths during transport with whole carcasses condemned in the present study, which is in fact perfectly in agreement with the rate found by the aforementioned study [51]. Differences in the approach to the collection, analysis, and presentation of data between the various studies represent an issue for their comparison, in addition to other factors discussed below (Section 4.5). In addition, specific commercial agreements between the slaughterhouse and the farms can influence this aspect. In fact, in the analyzed slaughterhouse, only the carcasses fit for human consumption are paid to the owner.

As for other European countries, a higher figure (0.24%) has been published for Portugal by Garcia-Diez et al. [30], while data for the United Kingdom is even higher; according to estimates by the Food Standards Agency, in England and Wales, 3 carcasses per 1000 slaughtered pigs were condemned for pathological alterations in 2015, to which an additional half carcass per 1000 animals for process-related defects must be added, for a total loss of 0.35%, a value that is reported as stable over 10 years [55].

The causes of whole carcass condemnation found in this study are reported in Table 2. Although erysipelas was the first cause, the absolute percentage of cases found in this study is very low (0.01%), lower to what was found in another Italian study conducted by Ghidini et al. [24], in which the disease was diagnosed in 0.3% of the pigs examined. On the contrary, no cases were found in the study of Ceccarelli et al. [22]. The percentage observed in this work is lower than that reported in a retrospective study on the causes of condemnation in pigs slaughtered between 2002 and 2016 in the Southeast of Spain (0.074) [50]. The disease has also been reported with varying percentages in slaughtered pigs in Portugal [30] and Switzerland [56]. Swine erysipelas is influenced by various factors [57], among which sudden changes in environmental temperature are particularly important [50,57]. Although the causes of skin erythema in pig carcasses are multiple, including incorrect husbandry and/or stress, inappropriate exsanguination at slaughter, and generalized infectious disease [14], classical erysipelas cutaneous lesions are characteristic and a study conducted in the US showed that cases of suspected swine erysipelas condemned at an abattoir were always appropriately classified [58]. Considering the scarce updated epidemiological data regarding this disease in Italy, seasonal and geographical trends will be investigated in a dedicated article.

Although generalized jaundice was the second cause of carcass condemnation, its percentage on slaughtered pigs (0.007%) was much lower than the percentage of 0.09% observed by Maisano et al. [51], a work in which jaundice was the first cause of condemnation, and also of the percentage of 0.04% of another recent Italian study [24], while it was more similar to the finding of Portuguese authors (<0.01% [30]). Several causes may be responsible for this condition in pigs, including viral (e.g., circovirosis), bacterial (e.g., leptospirosis), and parasitic (e.g., ascaridosis) infections, as well as intoxications [59,60]. However, these cases were not further investigated using laboratory analysis. It has to be remarked that updated information on the health status of the pig population in a given area are of particular importance also in consideration of the growing spread of African swine fever in previously free countries, including Eastern Europe [61].

Lipomatous pseudohypertrophy, a dystrophic condition of muscle tissue with adipose tissue infiltration, is not reported in the recent bibliography and in our study appears limited to the first six years examined, probably due to a genetic defect of the reproducers (authors’ personal communication).

As regards the other causes of carcass condemnation (Table 2), these are in agreement with those reported in similar studies; abscesses were the first cause of whole carcass condemnation in Switzerland, as they were responsible for 35% of the condemnations [56] and were frequently reported also in Italy [22], as well as in studies conducted in Third countries [41]. Among other causes, peritonitis, septicemia and cachexia [22]; fractures/hematomas and peritonitis [41]; and pleurisy/pneumonia and peritonitis [30] have been described. Although taken individually these causes appear to be of small relevance, it should be noted that altogether they account for the condemnation of 99 pigs, corresponding to 26.6% of the total condemnations, thus equaling the second cause (generalized jaundice).

### 4.4. Partial Condemnations

The analysis of partial condemnations was focused on the four most frequently condemned organs: liver, lung, heart, and kidney (Table 3), which will be discussed below and compared with the available literature, although several studies excluded partial condemnation data from their analysis [30,41,44]. Liver condemnations were the most frequent, as already observed in another study conducted in central Italy [22]. The main cause is by far the so-called “milk spot liver”, deriving from the larval migration of *Ascaris suum* [62] and responsible for 91.79% of the condemnations. This is in agreement with most of the studies in the literature in Italy [22,24,51] and in other European countries, such as Portugal [63] or Northern Ireland [64,65]. The frequent finding of this lesion confirms that ascaridosis is still a widespread disease and a potential cause of high economic losses, both for the reduced growth in the breeding phase and for the increased percentages of slaughterhouse discard [63,65,66]. Slaughterhouse output can be used to verify the application and effectiveness of control programs and anthelmintic protocols [67]. With reference to this, according to the official veterinarians involved in the inspection of the slaughterhouse examined in this work, famers may choose to avoid antiparasitic treatments considering the low market value of the pig liver in Italy. Monitoring the presence of *A. suum* can also have a public health value. In fact, hybrids of *A. suum* and *A. lumbricoides* (the species responsible for human ascaridosis) can be responsible, although rarely, for human infestation. A case in Italy was reported in a pig farmer in the province of Cuneo by Dutto et al. (2013) [68].

As for the other causes of liver condemnation, perihepatitis is involved in almost 7% of the condemned livers. This lesion can be due to infectious polyserositis, attributable to serious infections with *Actinobacillus pleuropneumoniae* or other primary polyserositis agents, or be secondary to lung infections [67]. Therefore, a specific diagnosis is not possible when slaughtering. In particular, the absence of further investigations does not allow hypotheses regarding the etiology of the lesions found in this study.

The main causes that led to lung condemnations are attributable to pneumonia and pleurisy. Lesions from enzootic pneumonia, characterized by cranio-ventral consolidation and caused by numerous bacteria, in particular *Mycoplasma hyopneumoniae* and *Pasteurella multocida*, and pleurisy, whose main causative agent is *A. pleuropneumoniae*, are reported as the most frequent findings at the pig slaughterhouse [69], albeit with very variable percentages. In the study by Ghidini et al. [24] a prevalence of 6.43% was observed for pneumonia, which is therefore higher than that found in our work (for lesions indicated exclusively as pneumonia the prevalence was in fact 3.5%), and a prevalence of 15.46% for pleuropneumonia, which is also higher than our 6.9% for this specific lesion. Even higher values are reported in heavy pigs slaughtered in Northern Italy (17% pneumonia and 26% pleurisy [51]; 46.4% bronchopneumonia, 47.5% pleurisy [69]) and also for domestic slaughtering in Friuli and Piedmont, in which lung lesions were the main cause of condemnation (65% [70]). On the contrary, in the study of Ceccarelli et al. [22] lungs were condemned only in 0.12% of the animals and the lesions were attributed almost exclusively to polyserositis. The observed variations may be due to differences in the inspection judgment or may also be attributed to the specific purposes of the research work if these overlap with the normal inspection activity [24].

Heart condemnations were mainly due to pericarditis, an unspecific lesion that has been associated with bacterial infections, in particular *Haemophilus parasuis*, *Pasteurella* spp., *Mycoplasma* spp., and *Streptococcus* spp. [65]. The observed rate is comparable to the one reported by Maisano et al. [51] in Italy (4.25%) and is slightly higher than that reported by Ghidini et al. [24] in Italy (3.22%) and by Correia-Gomes et al. [65] in Northern Ireland (3.5%). In the study of Ceccarelli et al. [22] the heart was the second most condemned organ (10.77% of the slaughtered pigs), almost exclusively due to pericarditis (observed in 99.8% of the condemned hearts). The second cause of heart condemnation was polyserositis, which has already been mentioned in relation to the lung, in accordance with the fact that it is a known cause of condemnation of the thoracic organs and sometimes also of the liver [67].

As for kidneys, the main pathological lesions observed were polycystic kidney and nephritis. In this case, the comparison with literature data is complicated by the non-uniform lesion classification. Ghidini et al. [24] report a prevalence of less than 0.3% for nephritis, but also a similar value (0.3%) of nephrosis. In the study carried out by Ceccarelli et al. [22], the number of seized kidneys was lower than 0.06% of the total, clearly lower than our value, but the condemnation causes are not indicated.

### 4.5. Meat Inspection in Pig Abattoirs: The Need for a Harmonized Data Collection System

Despite the will of the European legislator to standardize MI over the entire European Community, differences have already been reported [12]. In fact, the specific training, experience, and motivation of each official veterinarian, as well as operational aspects of slaughter (line layout, speed and number of inspectors), the classification of the lesions found and the data collection system inevitably influence the MI output [12,13,71] in different slaughterhouses, regions, or European nations [11,24,67], although MI is conducted following the same legal basis (in particular, from 2006 to 2019 following Reg. CE 854/2004 and currently according to the Reg. (EU) 2017/625 of the European Parliament and of the Council [7] and the Commission Implementing Reg. (EU) 2019/627). In addition, a univocal registration system for the collection of inspection data at the European level is still missing [13,71]. The animal category also represents a variability factor; in Italy, most of the animals bred and slaughtered are “heavy” pigs, while the European farming system generally produces “light” pigs (about 110 kg live body weight) [5,47]. Finally, among the published studies, some, like the present work or for instance the one of Ceccarelli et al. [22], have analyzed inspection data deriving from official veterinarians’ activities, while others have instead envisaged additional inspections and/or sampling for specific data collection or for the development of alternative inspection protocols [24,72]. All these factors can limit the data comparison [13,67,71], which is known to be challenging [15]. Limitations in the reliability of inspection data have also been described and need to be considered [71].

In general, Member States should be required to submit an annual report to the Commission with information on control activities [7]. As for the meat chain specifically, the CA shall enter the results of official controls in relevant databases, at least where the collection of such information is required under Article 4 of Directive 2003/99/EC (Art. 39 [16]). Implementing regulations are in fact used where it is necessary to ensure uniform conditions of implementation within the Union even on topics which normally fall within the competence of the Member States. In fact, a proper data flow towards organizations dealing with risk assessment will allow a continuous revision of hazard identification and ranking, taking into account EU regional variability. At the same time, these data may be useful for farm and abattoir risk categorization, assessing the efficacy of inspection procedures and eventually developing alternative control measures. Therefore, the production of scientific results will enable the policy makers to identify measures for reduction of the public and animal hazards [15]. Appropriate and dedicated training should be provided to promote a uniform approach to official controls and other official activities by the CA [7]. In this light, initiatives such as the Slaughterhouse Support Network (SESC), implemented by the Catalan Public Health Protection Agency with the main goal to offer continuing education to meat inspectors to improve the diagnostic capacity for lesions observed in slaughterhouses [14] should be supported and replicated. Furthermore, optimization and harmonization of the collection and communication of inspection results, with specific reference to the information sent backwards to farms regarding MI, represent one of the currently ongoing topic of the RIBMINS COST Action [15]. Although EU legislation (Regulation EC 853/2004 [73]) introduced the FCI concept more than a decade ago, original intentions on its use have largely not been achieved, and to date, it has been underdeveloped and underutilized [15]. Interestingly, a communication failure between CA and farmers has been hypothesized by other authors in the case of a stationary trend of the main diseases over the years [22], which was observed also in this study. In Britain, it was observed that companies that paid attention to the feedback received from the slaughterhouse improved their scores over time, presumably associated with improved measures in disease management [50].

In Italy, this approach has been recently advocated in the Classyfarm system, funded by the Ministry of Health and implemented by the Istituto Zooprofilattico Sperimentale of Lombardy and Emilia Romagna (IZSLER) with the collaboration of the University of Parma [31]. Classyfarm is proposed as a voluntarily risk categorization system for farms and allows the collection and processing of various data, including lesions detected at the slaughterhouse. The system is part of the national veterinary portal [34] and is available to official veterinarians, farm veterinarians and farmers, to comply with and fully implement the recent European legislation on Animal Health Law [74] and on official controls [7,16]. This system should promote a process of amelioration of the farming practice, improving production and reducing drug use and slaughterhouse lesions.

## 5. Conclusions

Despite the issues deriving both from intrinsic (lesion classification and data collection system) and extrinsic (different approaches between the various studies and scarcity of studies in the literature) factors, the analysis of whole carcass and partial condemnations is a valid tool in the study of the type and prevalence of lesions found in slaughtered animals. In particular, the analysis of data from the MI activity at the slaughterhouse is a useful instrument to analyze the trend of the main diseases over time and the results of the efforts for their control, and also to monitor compliance with animal welfare standards. Overall, the results of this study describe a non-worrying situation as regards the investigated aspects. The mortality rate was found to be lower than the threshold internationally recommended to ensure animal welfare and in line with the mortality values reported at the European level. As regards whole carcass condemnations, mainly caused by erysipelas and generalized jaundice, the low percentage was constant over the years and, as expected, significantly lower than partial condemnations. These principally affected liver, lungs, heart, and kidneys. Liver condemnations due to “milk spot liver” were by far the most frequent, confirming that ascaridosis is still a widespread disease. Although an integrated approach throughout the supply chain is necessary to ensure public and animal health, and keeping the harmonization limits for collection and data comparison in mind, the MI conducted at the slaughterhouse by the official veterinarians continues to play a fundamental role. The slaughterhouse can still act as an epidemiological observatory to provide the data needed for the development of plans for the control and eradication of the most frequent diseases in the area, also considering that this kind of surveillance is more cost-effective for many pathologies than the collection of data on farms.

## Figures and Tables

**Table 1:** Details of slaughtered pigs, deaths during transport, and condemned whole carcasses per year and for the total period (2010–2019) including number and absolute and relative percentage.

Year	*n* Slaughtered Pigs	Pig Deaths during Transport			Whole Carcass Condemnations		
		*n*	Absolute ^a^ %	Relative ^b^ %	*n*	Absolute ^a^ %	Relative ^c^ %
2010	113,368	293	0.26	25.41	45	0.04	12.10
2011	117,686	258	0.22	22.38	37	0.03	9.95
2012	120,720	22	0.02	1.91	44	0.04	11.83
2013	118,183	19	0.02	1.65	31	0.03	8.33
2014	120,849	206	0.17	17.87	36	0.03	9.68
2015	145,489	119	0.08	10.32	34	0.02	9.14
2016	126,015	59	0.05	5.12	38	0.03	10.22
2017	127,980	63	0.05	5.46	34	0.03	9.14
2018	130,174	73	0.06	6.33	37	0.03	9.95
2019	125,845	41	0.03	3.56	36	0.03	9.68
2010–2019	1,246,309	1153	0.09	-	372	0.03	-

^a^ Percentage calculated on the total number of pigs conferred to the slaughterhouse (total number of slaughtered pigs plus pigs that died during transport) per year and for the 10-year period; ^b^ percentage calculated on the total number of pigs that died during transport in the 10-year period; ^c^ percentage calculated on the total number of condemned whole carcasses in the 10-year period.

**Table 2:** Details of the frequency and causes of whole carcass condemnations. PSE: pale soft exudative.

	2010	2011	2012	2013	2014	2015	2016	2017	2018	2019	*n*	Absolute ^a^ %	Relative ^b^ %
Erysipelas	10	16	7	11	11	11	26	20	6	21	139	0.0111	37.36
Generalized jaundice	5	4	14	4	10	14	8	8	22	8	97	0.0078	26.07
Lipomatous pseudohypertrophy	8	8	14	5	1	1					37	0.0030	9.95
Abscesses	3	3	2	7	3	5	2	1	1	1	28	0.0022	7.53
Enteritis	6	3	3	-	1	-	1	2	1	3	20	0.0016	5.38
Peritonitis	1		1		2	2	1	1	5	1	14	0.0011	3.76
Cachexia	3	1	2	1	2	-	-	-	-	1	10	0.0008	2.69
Pleuritis	8										8	0.0006	2.15
Errors in the slaughtering process	1			1				1	2		5	0.0004	1.34
Perihepatitis					4						4	0.0003	1.07
Traumatic lesions								1		1	2	0.0002	0.54
Neoplasia					1	1					2	0.0002	0.54
Polyserositis				1	1						2	0.0002	0.54
Disseminated hemorrhagic syndrome		1		1							2	0.0002	0.54
PSE		1									1	0.0001	0.27
Septicemia			1								1	0.0001	0.27
Total	45	37	44	31	36	34	38	34	37	36	372		

^a^ Percentage calculated on the total number of pigs conferred to the slaughterhouse (total number of slaughtered pigs plus pigs that died during transport) for the 10-year period; ^b^ percentage calculated on the total number of condemned whole carcasses in the 10-year period.

**Table 3:** Details of the frequencies of the most condemned organs per year and for the total period (2010–2019). *n*: number of organs.

	Liver		Lungs		Heart		Kidney	
Year	*n* ^a^	Absolute %	*n*	Absolute %	*n*	Absolute %	*n*	Absolute %
2010	25,866	22.76	22,600	19.88	8337	7.33	712	0.63
2011	33,381	28.30	21,825	18.50	8904	7.55	837	0.71
2012	30,541	25.29	22,062	18.27	9495	7.86	804	0.67
2013	35,400	29.95	18,340	15.52	12,206	1033	968	0.82
2014	47,656	39.37	17,414	14.38	10,217	8.44	981	0.81
2015	56,004	38.46	25,911	17.79	8708	5.98	1558	1.07
2016	35,324	28.02	13,987	11.09	5649	4.48	2072	1.64
2017	35,994	28.11	18,132	14.16	6445	5.03	1166	0.91
2018	40,069	30.76	20,603	15.82	8031	6.17	1333	1.02
2019	44,774	35.57	34,444	27.36	8564	6.80	1366	1.08
2010–2019	385,009	30.86	215,318	17.26	86,556	6.94	11,797	0.95

^a^ Percentage calculated on the total number of slaughtered pigs.
